# Different Methods of Resection of Solid Pseudopapillary Neoplasm of the Pancreas: A Case Series of Three Patients

**DOI:** 10.7759/cureus.7346

**Published:** 2020-03-20

**Authors:** Samriddha R Pant, Nishan B Pokhrel, Parikshit Chapagain, Prasan Kansakar

**Affiliations:** 1 Surgery, Tribhuvan University Institute of Medicine, Kathmandu, NPL; 2 Internal Medicine, Tribhuvan University Institute of Medicine, Kathmandu, NPL

**Keywords:** cystic neoplasm, solid pseudopapillary neoplasm, pancreas, surgical resection

## Abstract

Solid pseudopapillary neoplasm of the pancreas is one of the rarest forms of pancreatic neoplasm. It was also known as Franz's tumor or Hamoudi tumor until the World Health Organization (WHO) labeled it as a solid pseudopapillary tumor in 1996. It typically affects young non-Caucasian females in their second or third decade of life. Treatment involves complete excision of the tumor which results in a complete cure in most of the cases. Three cases of solid pseudopapillary neoplasm (diagnosis confirmed by cytology) in young females, each presenting with different symptoms were studied. Each of the three cases was found to have the neoplasm at different sites of the pancreas and was subjected to different resection procedures. The cases were followed up for at least a year and evaluated for recurrences/metastases. Solid pseudopapillary neoplasm remains one of the most misdiagnosed tumors. The diagnosis depends on radiology and cytology. With a very high five-year survival rate, surgical resection remains the treatment of choice. The type of surgical procedure depends on the site, size and local invasion of the tumor.

## Introduction

Solid pseudopapillary neoplasm (SPN) of the pancreas is a rare tumor accounting for approximately 0.2% to 2.7 % of primary non-endocrine tumors of the pancreas. These tumors have low malignant potential and a strong predilection for young women, with less than 10% of cases reported in men [1]. In 1996, the World Health Organization (WHO) defined it as a solid pseudopapillary tumor of the pancreas. Before this nomenclature, it was known by many names, including Frantz’s tumor, solid and papillary tumor, solid-cystic tumor, papillary cystic tumor, and solid and papillary epithelial neoplasm. Distant metastasis is seen in 10%-15% of these tumors and mortality is reported in 2% of all cases [2].

SPN is an epithelial neoplasm consisting of discohesive polygonal cells that surround delicate blood vessels, which form a solid mass. It also consists of pseudopapillary structures formed by morphologically consistent cells. Areas of hemorrhage and cystic degeneration are also present in these neoplasms. These cystic parts result from the degeneration of pre-existing solid components [3]. Although the etiopathogenesis of these neoplasms remains uncertain, its occurrence in young women at the beginning of the reproductive period, as well as the presence of progesterone receptors in it, points towards the role of female sex hormones in its growth [4]. Chromosomal mutations may also have an etiologic role for these neoplasms. As suggested by some studies, a mutation in beta-catenin could play a major role in tumor development [5].

Here we present three cases, which occurred at three different sites of the pancreas and were resected using different surgical methods.

## Case presentation

Case 1

A 20-year-old female presented to the gynecology outpatient clinic with an acute onset lower abdominal pain during her menses along with heavy vaginal bleed. At the time of presentation, she had mild pain which occasionally radiated to the umbilical region. On general examination, the patient had mild pallor. On deep palpation of the abdomen, she had mild tenderness in the hypogastrium.

**Figure 1:**
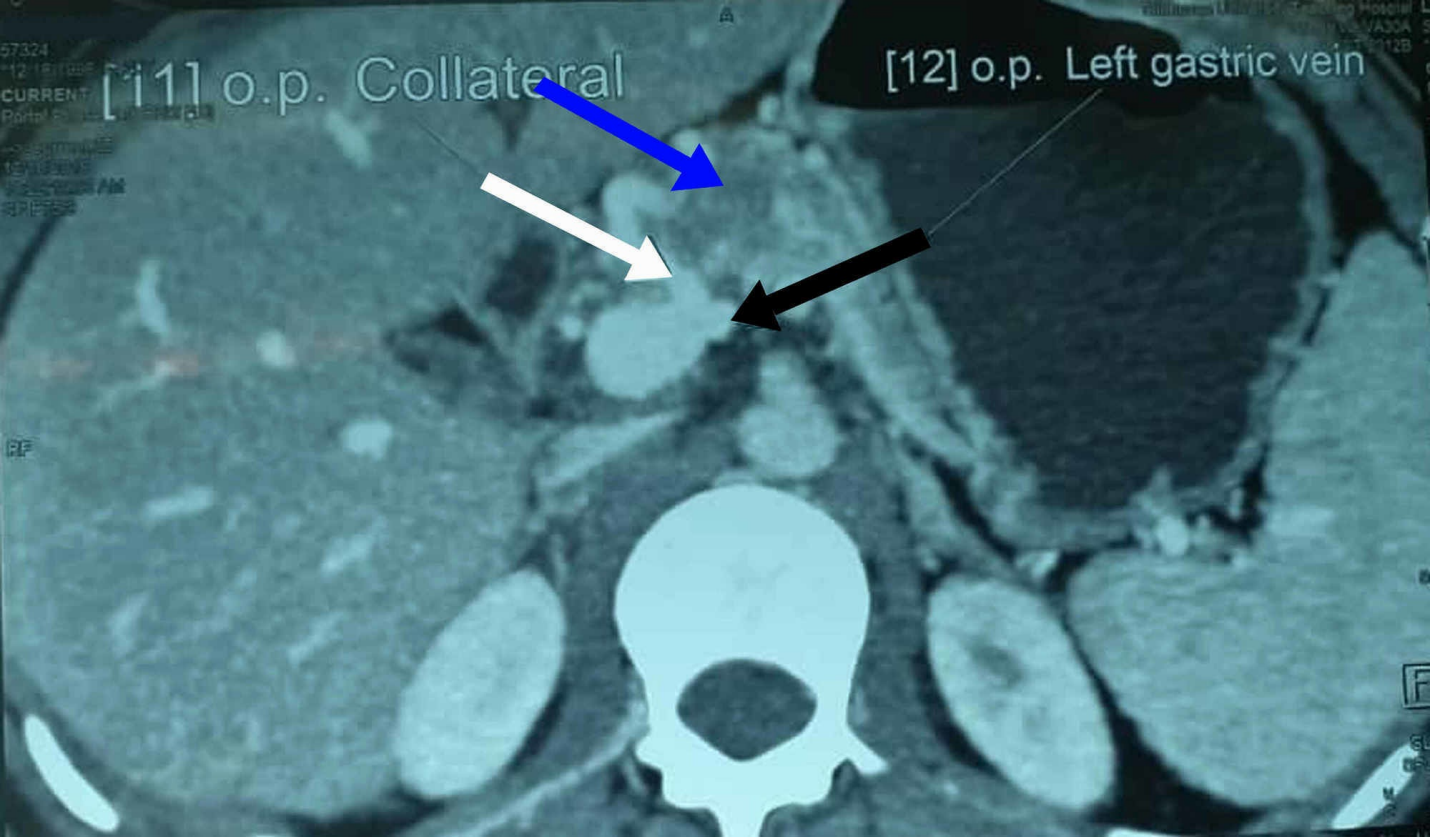
CECT showing the mass in the neck and body of the pancreas with solid/cystic areas (blue arrow), left gastric vein (black arrow), and a dilated collateral vein arising from inferior vena cava (white arrow) CECT, contrast-enhanced computed tomography

Ultrasonography (USG) of the abdomen and pelvis revealed no significant findings in the reproductive tract. A contrast-enhanced computed tomography (CECT) scan of the abdomen showed a 2 cm X 3 cm sized solid mass in the body and neck of the pancreas (Figure 1). Preoperative blood and urine examinations were normal except for hemoglobin, which was 9.4 g/dL (normal range in female: 12-16 g/dL).

**Figure 2:**
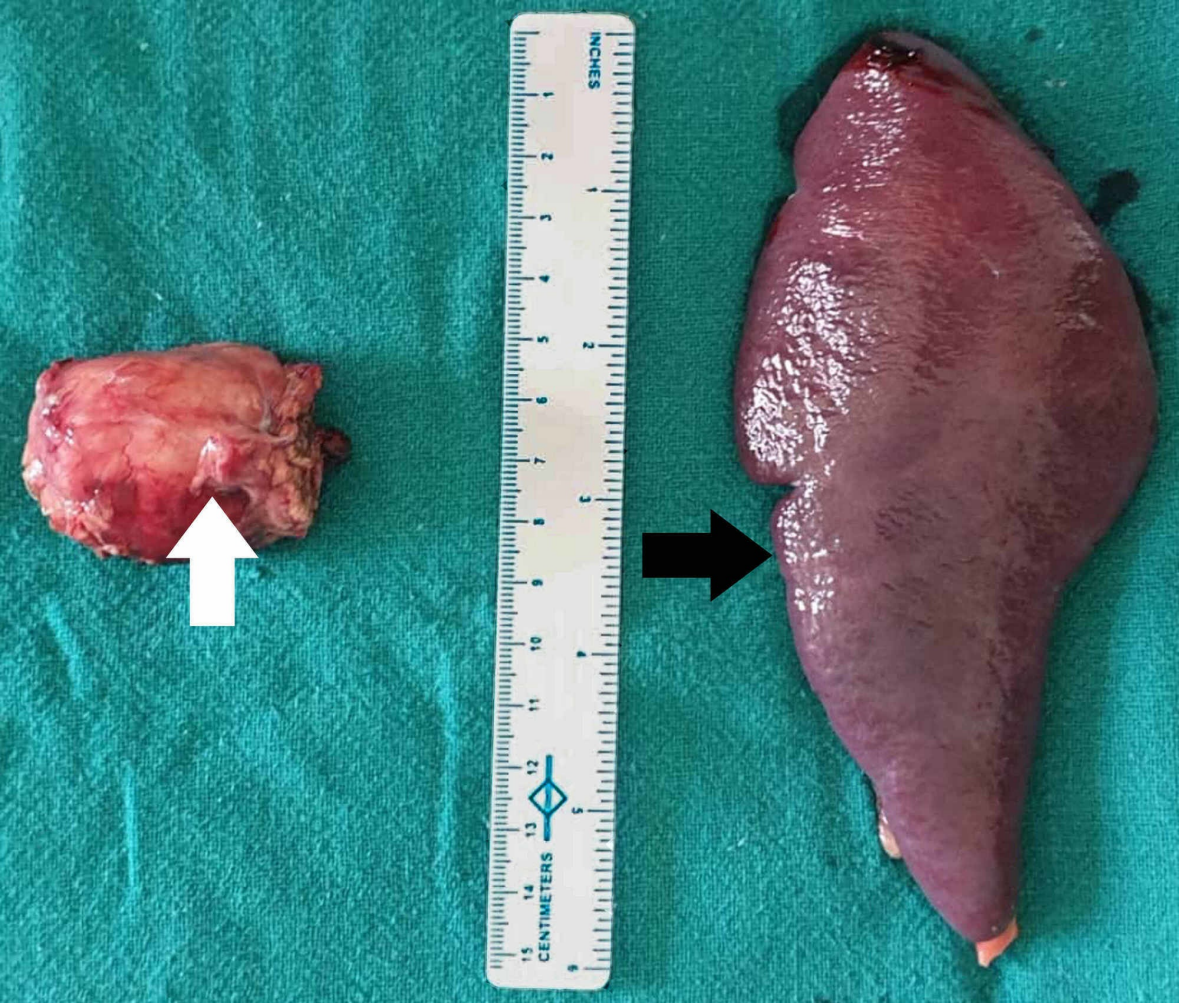
Resected specimen showing central pancreas with the mass (white arrow) and the spleen (black arrow)

On exploratory laparotomy, a 2 cm X 3 cm mass was seen in the body of the pancreas. The mass was seen compressing the splenic vein causing its complete obliteration. Multiple collateral vessels were seen around the tumor as sequelae of splenic vein thrombosis. The portal vein was free from the tumor. A central pancreatectomy (Figure 2) with splenectomy was performed. Splenectomy was performed as the patient had an occluded splenic vein and collateral vessels to the spleen, and also had sinistral portal hypertension. After ligation of the splenic artery, the collaterals decreased as outflow from spleen decreased. Hence, central pancreatic resection could be performed with around 6 cm distal pancreatic stump left. The postoperative period was uneventful. She was discharged on her ninth postoperative day. Histopathological examination showed mostly solid areas alternating with a cystic background, suggesting a SPN. She was followed up for two more weeks during which she was recovering normally and no issues were found. On a 12-month follow-up period, she had no signs and symptoms of recurrences or metastasis.

Case 2

A 31-year-old female presented with the symptom of lower abdominal pain for the past eight months. She had normal menstrual history. On examination, her abdomen was soft with mildly tender left hypochondrium.

**Figure 3:**
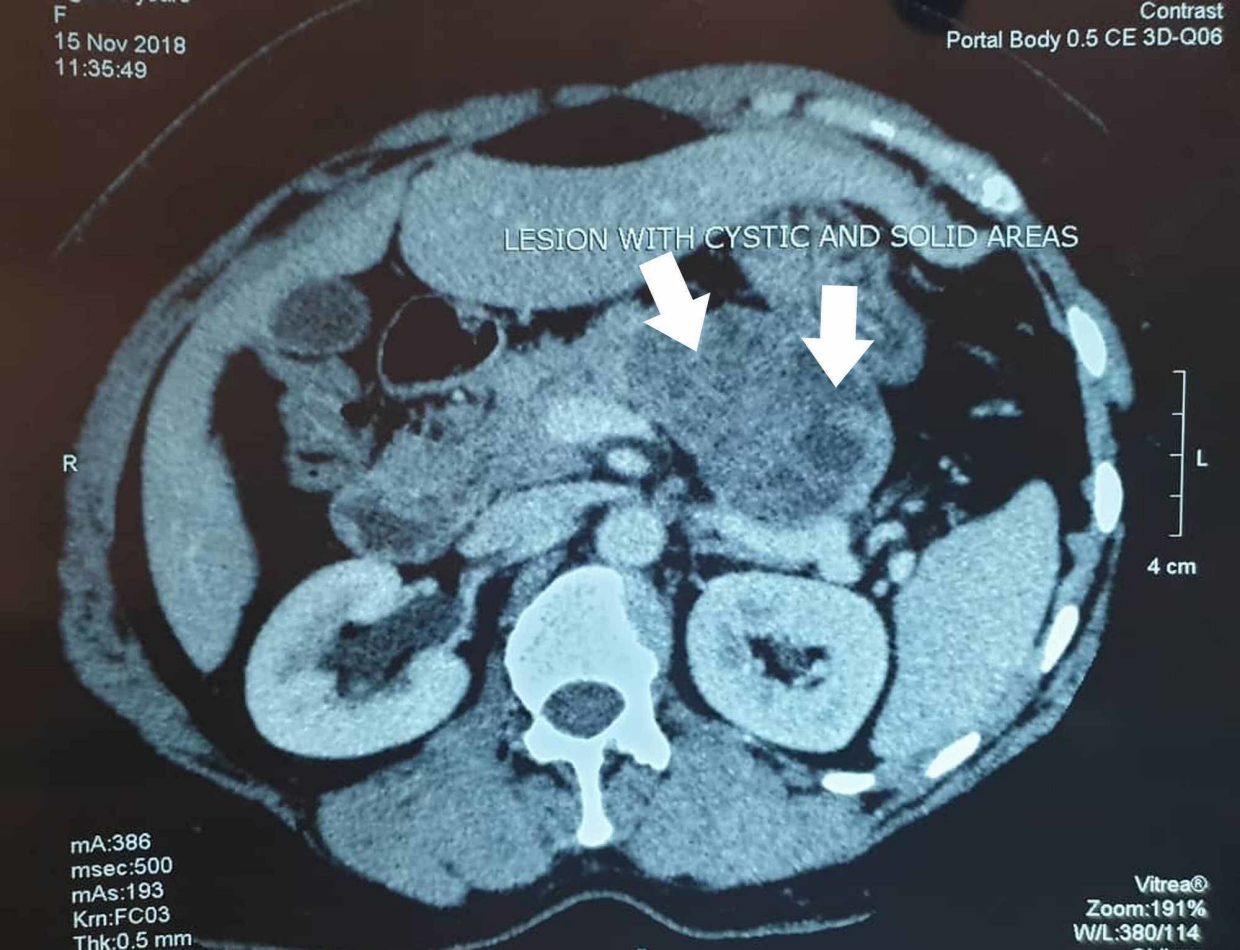
CECT showing the mass in the body and tail of the pancreas with solid areas alternating with cystic spaces (white arrows) CECT, contrast-enhanced computed tomography

USG of the abdomen showed a 5 cm X 6 cm hyperechoic mass in the body and tail of the pancreas. CECT scan of the abdomen showed a 5 cm X 6 cm lesion arising from the pancreas with solid and cystic areas (Figure 3). The radiologic scans did not show spread of the tumor elsewhere. Preoperative blood and urine examinations were normal.

**Figure 4:**
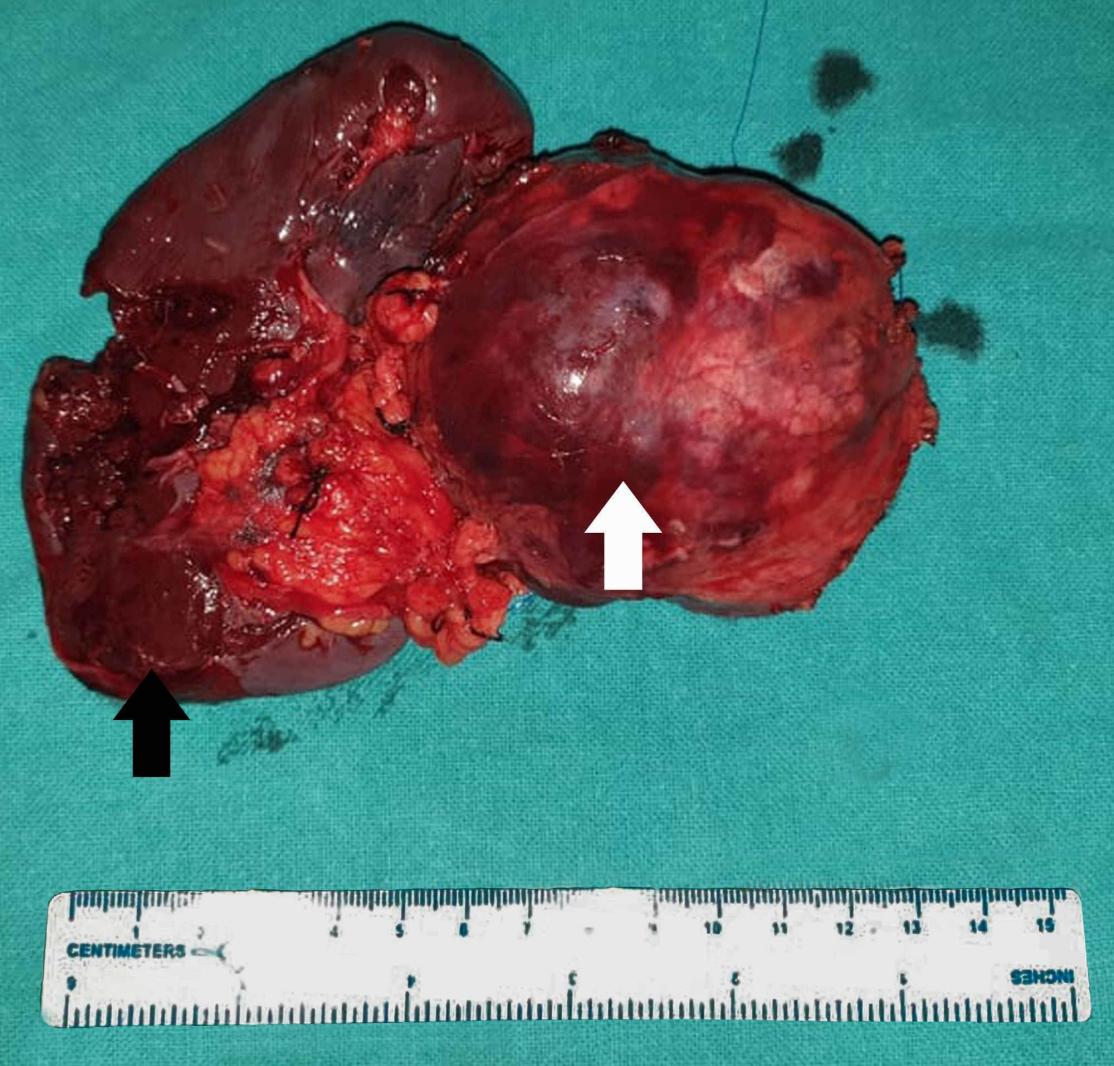
Resected specimen with distal pancreas (white arrow) and spleen (black arrow)

Preoperatively, a mass with solid and cystic components was seen arising from the body and tail of the pancreas. All the major vessels in close proximity to the tumor were free of invasion. Distal pancreatectomy (resection of body and tail of pancreas) with splenectomy (Figure 4) was performed. Splenectomy was done as the mass was relatively large and abutting the splenic vein. The nature of the lesion, whether benign or malignant, was also not known preoperatively as a preoperative biopsy could not be performed. Histopathology revealed solid areas with pseudopapillary projections, alternating with cystic spaces. She was discharged after a week of her operation and was followed up for 14 months. During follow-up, her symptoms resolved and no symptoms or signs of recurrence and metastases of the tumor were noted.

Case 3

A 22-year-old female presented with pain in the epigastric region for one and a half years. The pain was dull aching, and would wax and wane with time. It was unrelated to food intake, and water brash was absent. She also complained of nausea and anorexia over the past two months, and her bowel habit was normal. Swelling could be noted in the epigastrium, measuring 3 cm X 3 cm, smooth, firm in consistency, and tender on palpation.

**Figure 5:**
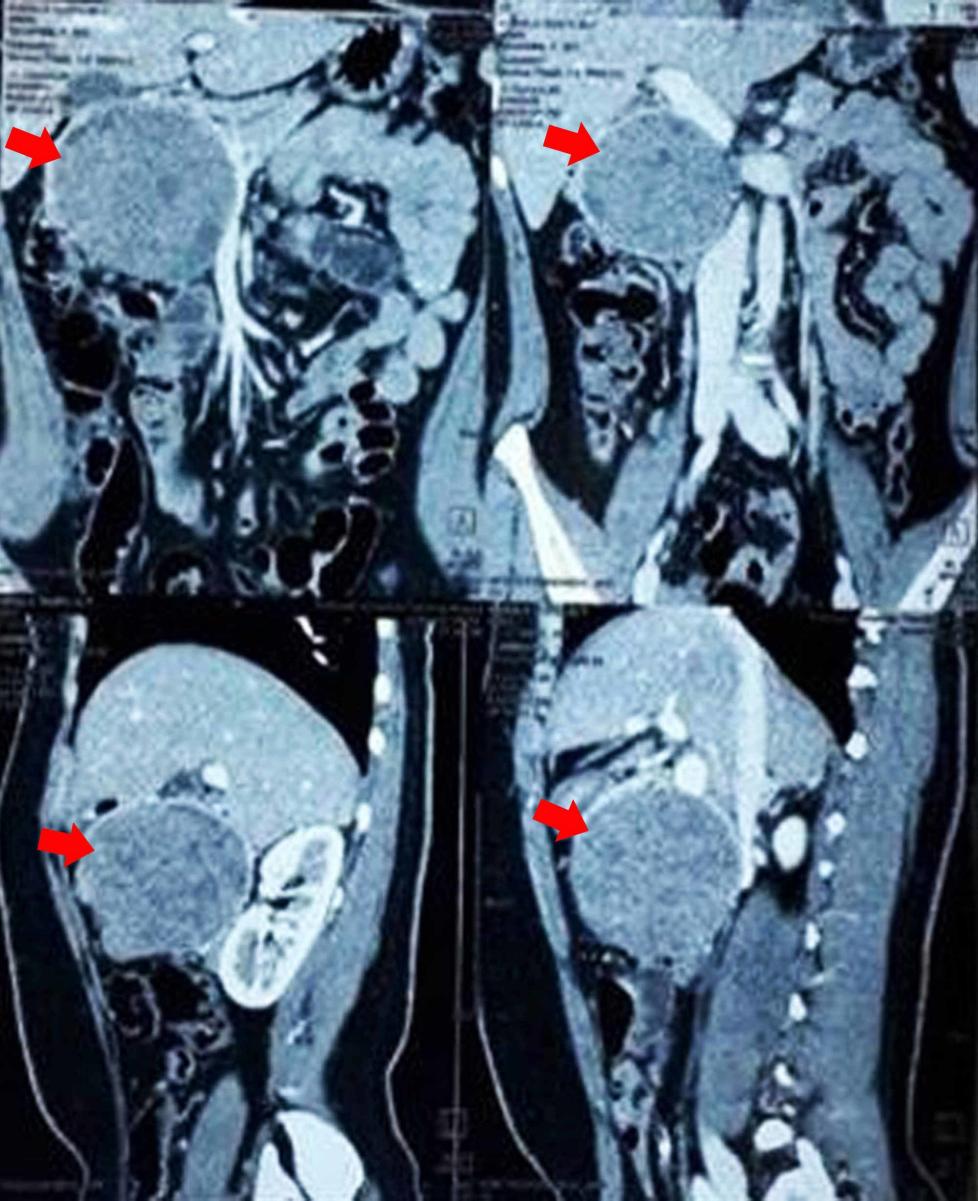
CECT showing mass in the pancreatic head (red arrows) CECT, contrast-enhanced computed tomography

USG of the abdomen revealed an 8 cm X 7 cm hypoechoic mass arising from the pancreas. CECT scan of the abdomen and pelvis showed an 8 cm X 7 cm mass in the head of the pancreas (Figure 5). All other preoperative investigations were normal. Whipple’s operation was planned.

**Figure 6:**
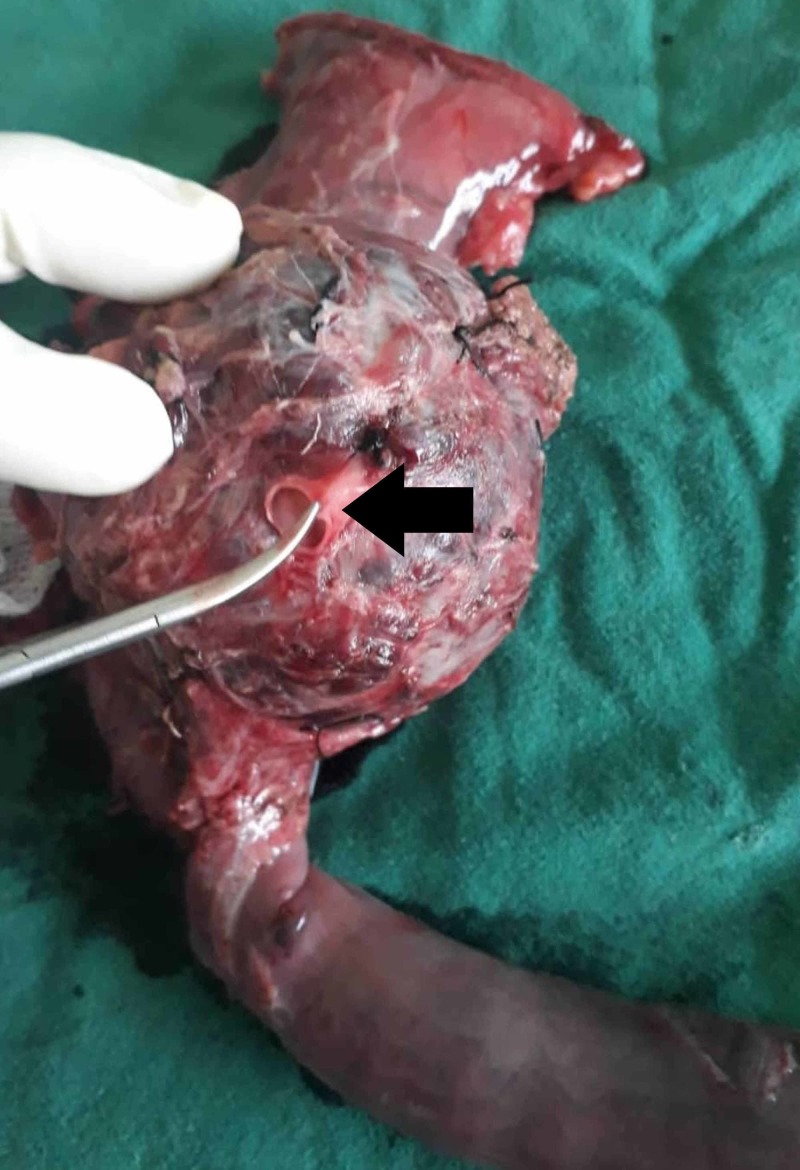
Resected specimen with portal vein (black arrow)

Intraoperatively, an 8 cm X 8cm tumor was noted arising from the head of the pancreas. The mass could not be separated from the lateral wall of the portal vein. Hence, portal vein resection (Figure 6) with end-to-end anastomosis was done. Histopathology showed solid and cystic areas within the mass with necrosis and hemorrhage, suggesting a SPN. She was discharged after eight days of uneventful postoperative period and was followed up for 21 months. During this period, she did not show symptoms or signs of recurrence. She was tolerating a normal diet, and her quality of life was unaffected.

Table 1 summarizes the findings of all three cases.

**Table 1:** Summary of the three cases presented in this report

	Case 1	Case 2	Case 3
Age/sex	20 years/female	31 years/female	22 years/female
Presenting symptom	Incidental (while evaluating for dysmenorrhea)	Chronic lower abdominal pain	Pain and swelling in the epigastric region for one and a half years
Location	Body and neck of the pancreas	Body and tail of the pancreas	Pancreatic head
Size	2 cm x 3 cm	5 cm x 6 cm	7 cm x 8 cm
Surgical procedure	Central pancreatectomy with splenectomy	Distal pancreatectomy with splenectomy	Whipple’s operation with portal vein resection
Recurrence/metastases	None	None	None
Follow-up	12 months	14 months	21 months

## Discussion

More than 90% of SPNs have been reported in women under the age of 35 years, with a female to male ratio of 20:1 and an average age of incidence of 28 years [6]. The most common sites of tumor incidence within the pancreas include pancreatic tail and the head of the pancreas, followed by the pancreatic body, body and tail, head and body, neck, and the uncinate process [6]. The cases studied above remain consistent with regards to sex and age predilection, as the affected individuals were all females below the age of 35 years. Two of the three cases, however, had tumors arising from the pancreatic body with only one case affecting the pancreatic head.

There is no specific symptom complex that is related solely to these tumors of the pancreas, and due to their rarity, they often tend to be misdiagnosed [1]. The most common symptoms associated with them are abdominal pain, abdominal distension, and features related to gastric outlet obstruction due to pressure effect on the adjacent organs. In almost 30% of the cases, patients are asymptomatic [1]. Similar findings could be noted in the above-mentioned cases. In the first case, the tumor was an incidental finding while evaluating for dysmenorrhea. Chronic lower abdominal pain was the main complaint in the second case, while in the third case, epigastric pain and features of dyspepsia were seen.

Radiologic imaging and cytology form the basis of the diagnosis of these tumors. In CT scans, these tumors show no enhancement of the cystic portions and slight enhancement of the solid portions in the arterial phase and marked enhancement in the portal venous phase [7]. USG-guided fine needle aspiration cytology is the best technique for preoperative diagnosis of SPN. It is also less invasive than surgery. It is more effective than CT in differentiating neoplastic from non-neoplastic cysts, and also identifies malignant pancreatic cysts [8]. Immunohistochemical staining and mutation analysis of the beta-catenin gene help to distinguish SPN from other tumors of the pancreas [9]. However, in limited-resource settings, cytology is most often followed by surgical resection of the tumors, as was done in our case [1]. 

Surgical resection is the treatment of choice for these tumors. Resection is warranted with negative surgical margins. Depending on the location of the neoplasm, the type of pancreatectomy is decided. Pancreatoduodenectomy is performed for neoplasms in the pancreatic head, distal pancreatectomy for those in the pancreatic body/tail, and a central pancreatectomy is performed if the neoplasm is in the proximal body of the pancreas [10]. Involved adjacent organs should be aggressively approached with the goal of obtaining negative margins [6]. Lymph node metastasis is rare in these tumors, and overall metastasis or tumor recurrence after resection occurs in 10%-15% of the cases [11]. While adjuvant chemo/radiotherapy is reported to have had some success in cases of unresectable tumors, it still has an uncertain role in cases like this due to the limited experience of its use [6,11]. Different surgical procedures according to the site and local invasion of the tumor were used in the above-mentioned cases. The case involving the body and neck of the pancreas underwent a central pancreatectomy with splenectomy, the one involving the body and tail underwent distal pancreatectomy with splenectomy, and the tumor involving the pancreatic head was resected using the Whipple’s operation along with portal vein resection.

The five-year survival rate in patients undergoing surgical resection approaches 97% [2-4,11]. These tumors have low malignant potential and favorable prognosis, although they have locally aggressive features [12]. The third case mentioned above had locally invaded the lateral wall of the portal vein, and thus portal vein had to be resected. Resection is not contraindicated in cases of recurrence, local invasion, and limited metastasis, and some patients with unresectable SPNs may also have a long survival time [13]. In a variable follow-up period, all three cases have had a recurrence-free outcome with little to no impact on the quality of life.

## Conclusions

The SPN of the pancreas is a rare low-grade malignant neoplasm mostly affecting young women. The presenting symptom may be vague, or the patient may even be asymptomatic and thus is often misdiagnosed. Clinical findings along with radiological imaging and cytology form the basis of its diagnosis. Surgical resection of the tumor remains the mainstay of its treatment. Different surgical methods are used to resect these tumors depending on the part of the pancreas involved, the size of the tumor, and the extent of its local invasion.
